# Older Adults’ Propensity for Community: Age Differences in the Effects of Closeness on Shared Positivity

**DOI:** 10.1093/geroni/igaf122.3984

**Published:** 2025-12-31

**Authors:** Sydni Adams, Joseph Mikels

**Affiliations:** DePaul University, Chicago, Illinois, United States; DePaul University, Chicago, Illinois, United States

## Abstract

Positivity resonance describes a connection involving shared positive affect and biobehavioral synchronization (Fredrickson, 2013), which improves one’s health (Major et al., 2018; Wells et al., 2021). Positivity resonance may also increase communal strength (e.g., going out of one’s way to meet others’ needs), which improves relationships (Mattingly et al., 2011). Though older adults’ social networks are comprised largely of close relationships (Moore et al., 2016), older relative to younger adults may be more likely to engage more distant others with positivity resonance, which may explain why weak ties are predictive of healthy aging (Huxhold et al., 2020). We explored the relationship between positivity resonance and communal strength for older and younger adults across strong, weak, no tie conditions. 250 participants (125 younger adults; 125 older adults) reported their feelings of positivity resonance and communal strength after recalling recent interactions across each level of tie strength. Using multilevel regression, we found an interaction of age and tie strength on positivity resonance, F(1, 468) = 1.38, p < .05, such that older adults experienced greater positivity resonance than younger adults for weak and no tie interactions. There was also an interaction between tie strength and positivity resonance on communal strength, F(1, 600) = 8.85, p < .01. The more positivity resonance participants experienced, the more communal strength they reported. This research advances our understanding of the quality of network diversity among younger and older adults. Our findings suggest that older adults have improved relationships with acquaintances and strangers compared to younger adults.

